# Simultaneous occurrence of hyperthyroidism and fistulizing Crohn’s disease complicated with intra-abdominal fistulas and abscess: a case report and review of the literature

**DOI:** 10.4076/1757-1626-2-8541

**Published:** 2009-08-25

**Authors:** Ioannis Pachiadakis, Andreas Nakos, Presvia Tatsi, John Moschos, Stefanos Milias, Panagiotis Nikolopoulos, Christos Balaris, Dimosthenis Apostolidis, Petros Zezos

**Affiliations:** 1 Department of Gastroenterology and Hepatology, 424 Military General HospitalThessalonikiGreece; 2 Department of Pathology, 424 Military General HospitalThessalonikiGreece; 3 Department of Radiology, 424 Military General HospitalThessalonikiGreece; 4 Department of Endocrinology, 424 Military General HospitalThessalonikiGreece; 5 Department of Surgery, 424 Military General HospitalThessalonikiGreece

## Abstract

**Introduction:**

Fistula formation in patients with Crohn’s disease is a common complication during the course of the disease. Perianal and enteroenteric are the most common forms of fistulas, whereas the involvement of the upper gastrointestinal tract with gastrocolic and duodenocolic fistulas represents an extremely unusual condition. Moreover, hyperthyroidism in association with Crohn’s disease has been rarely described.

**Case presentation:**

We present here a rare case of a 25-year-old male with simultaneous onset of hyperthyroidism and fistulizing Crohn’s disease. Crohn’s disease was complicated with intra-abdominal fistulas involving the upper gastrointestinal tract (duodenocolic, gastrocolic) and an intra-peritoneal abscess formation in the lesser sac. We describe the clinical presentation and therapeutic management of the patient including both medical treatment and surgical intervention. Despite intense medical treatment with total parenteral nutrition, antibiotics, aminosalicylates and corticosteroids the clinical course of the disease was suboptimal. Finally, the patient underwent laparotomy and right hemi-colectomy with ileo-transverse anastomosis performed, with simultaneous drainage of the abdominal abscess and primary closure of the upper gastrointestinal tract openings (gastric, duodenal and jejunal) at one stage operation. Although the surgical approach definitively cured the perforating complications of the disease (fistulas and abscess), the luminal disease in the colon remnant was still active and steroid-refractory. The subsequent successful treatment with infliximab, azathioprine and mesalazine resulted in the induction and maintenance of the disease remission. Thyrotoxicosis was successfully treated with methimazole and the hyperthyroidism has definitely subsided.

**Conclusion:**

The management of intra-abdominal fistulas in Crohn’s disease is a complex issue, requiring a multi-disciplinary approach and ‘tailoring’ of the treatment to the individual patient’s needs. Probably, a sensible approach involves early surgical intervention with prior optimization of the patient’s general condition when feasible. Common autoimmune mechanisms are probably involved in thyroid dysfunction associated with Crohn’s disease. Moreover, diagnosis and treatment of coexisting thyroid disorder in patients with Crohn’s disease has a favorable impact in disease prognosis.

## Introduction

Crohn’s disease (CD) is a chronic inflammatory disease of unknown etiology characterized by a chronic, granulomatous, segmental transmural inflammation that may affect any part of the gastrointestinal (GI) tract.

The transmural nature of inflammation in CD predisposes to the formation of fistulas. Fistulas are a common and often serious complication of CD, occurring in 30% to 50% of CD patients during the course of their disease [1].

In general, fistulas are classified as external and internal depending on their location and their connection with adjacent structures. External fistulas connect a diseased part of the bowel with the skin (enterocutaneous, parastomal and perianal fistulas), whereas internal fistulas connect either two parts of the bowel (enteroenteric, ileocolic, gastrocolic, duodenocolic) or a diseased part of the bowel and other adjacent organs (rectovaginal, enterovesical) [2].

Perianal fistulas are the most common type of fistulas in CD patients, followed by internal enteroenteric fistulas, while the involvement of the upper gastrointestinal tract with gastrocolic and duodenocolic fistulas is very rare and reported in few cases in the literature so far [1,3].

The coexistence of thyroid disease and inflammatory bowel disease (IBD) is uncommon. Thyroid dysfunction has been described more commonly in patients with ulcerative colitis (UC). On the contrary, its presence in patients with Crohn’s disease is extremely rare [4,5]. We report a rare case of simultaneous onset of hyperthyroidism and Crohn’s disease complicated with intra-abdominal fistulas (duodenocolic, gastrocolic) and an intra-peritoneal abscess formation in a 25-year-old male patient. We describe the management of the patient, exposing the therapeutic dilemmas that occurred, along with a review of the literature of those rare complications and associations.

## Case presentation

A 25-year-old, non-smoker, Caucasian male was admitted to our department with a 10-day history of vomiting, watery diarrheas, high fever (39^o^C) and crampy abdominal pain. His medical history was remarkable for hyperthyroidism due to a thyrotoxic goiter diagnosed 4 months before. However, it was left untreated and the patient mentioned a gradual body weight loss of 10 Kg during the last four months. His family history was unremarkable.

On admission the patient was febrile and looked severely ill. His height and weight were 186 cm and 95 Kg respectively (BMI 27.5 Kg/cm), and his vital signs included a temperature of 39^o^C, a blood pressure 130/80 mmHg, and a heart rate of 130 beats per minute. Physical examination revealed a mild to moderate tenderness at the epigastrium and the right upper abdominal quadrant. His thyroid gland was diffusely enlarged without tenderness. Laboratory data were remarkable for polymorphonuclear leukocytosis (WBC 17 × 10^3^/μl, neutrophils 77%), thrombocytosis (PLT 540 × 10^3^/μl), mild normocytic anemia (Hb 11.8 g/dl, MCV 88 fl), hypoalbuminemia (2.8 g/dl) and significantly elevated markers of inflammation (ESR 115 mm/1^st^ hr, CRP 126 mg/dl). Thyroid function tests included low thyroid stimulating hormone (TSH) (<0.03 μlU/ml; normal value 0.38-3.8 μlU/ml), high serum free T4 (FT4) (2.86; normal value 0.75-1.54 ng/dl) and normal serum free T3 (FT3) (3.7; normal value 1-3.8 pg/ml). Anti-thyroid antibodies were also elevated; the anti-thyroperoxidase antibody (anti-TPO) was 385.8 (normal value <50 IU/ml) and the anti-thyroglobulin antibody (anti-TG) was 88.5 (normal value <50 IU/ml).

**Figure 1.**
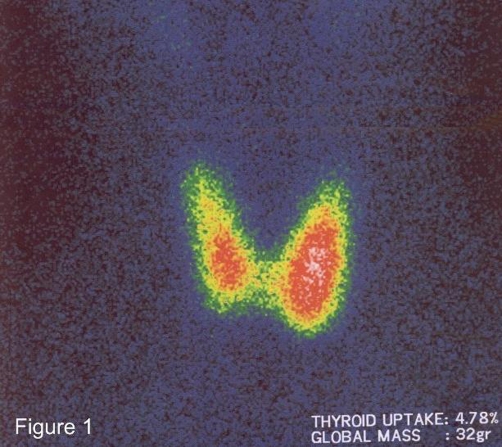
Thyroid Tc-99m scintigraphy. Diffusely increased homogenous localization of technetium pertechnetate (Tc-99m) in the thyroid gland. The gland looks enlarged, in particular the left lobe.

Ultrasonography of the thyroid gland revealed a multinodular goitre, whereas ^99m^Tc scan of the thyroid showed a moderate thyroid gland enlargement with increased thyroid gland total mass (32 gr; normal value <20 gr) and increased homogenous parenchymal uptake of technetium pertechnetate (4.78%; normal value 0.3-3%) (Figure 1). Treatment for thyrotoxicosis was promptly started with propranolol 20 mg tid and methimazole 10 mg tid.

**Figure 2.**
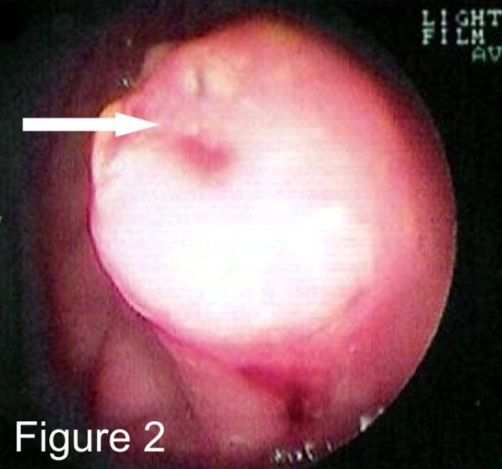
Gastro-duodenoscopy. A fistulous tract (arrow) opening on the greater curve at the antrum of the stomach with a crater on the top of an inflammatory process.

**Figure 3.**
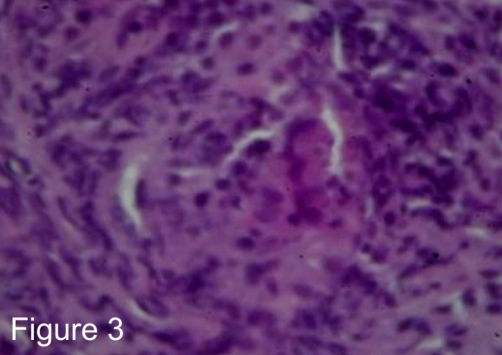
Histology. Histologic examination of a biopsy taken from the area of the fistulous tract opening in the stomach showing a non-caseating granuloma formation (H&E, ×400).

Soon after admission (2^nd^ day) the patient had bloody diarrheas without signs of hypovolemic shock and endoscopy of the upper and lower GI tract was performed. Colonoscopy to the terminal ileum demonstrated mild cecal inflammation with edematous, erythematous, non-friable and non-ulcerated mucosa without specific features on histology, while the upper GI endoscopy showed a normal esophagus, stomach and duodenum except for two fistulous tract openings, one in the greater curve of the stomach (Figure 2) and another in the second part of the duodenum. The histologic examination of the biopsies which were obtained from the fistulous openings showed non-caseating granuloma formation (Figure 3), while the random biopsies from the stomach and the duodenum were normal without features of inflammatory bowel disease.

**Figure 4.**
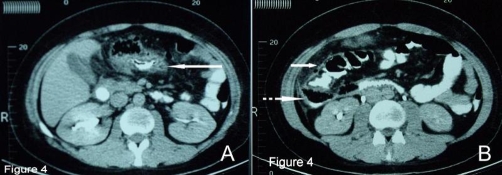
Computed tomography of the abdomen. **(A)** CT of the abdomen shows an ill-defined intra-peritoneal phlegmon in the lesser sac (continuous white arrow). **(B)** CT of the abdomen showing marked thickening of the wall of the ascending colon (continuous white arrow), air-fluid collection in the right anterior pararenal space admixed with gastrographin (dotted white arrow).

Computed tomography (CT) scan of the abdomen and pelvis showed an abscess formation without well-defined margins (phlegmon) in the lesser sac (Figure 4A), thickening of cecum, ascending and transverse colon wall and localized thickening of the omentum and mesentery (Figure 4B).

In the context of the findings above, treatment for severe active fistulizing Crohn’s disease (CDAI score: 452) complicated with an abdominal abscess was initiated with the institution of total parenteral nutrition (TPN) and the intravenous (IV) administration of high-dose prednisolone (75 mg/day), ciprofloxacin (400 mg bid), metronidazole (500 mg tid), and pantoprazole (40 mg bid), along with oral mesalamine (3 g/day), and azathioprine (2 mg/kg/day).

**Figure 5.**
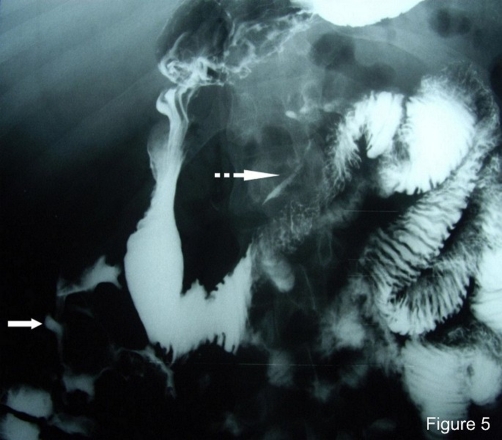
Small bowel barium follow-through study. Small-bowel barium follow-through study revealing fistulous tracts between the ascending colon and the 2^nd^ portion of the duodenum (continuous white arrow) and between the jejunum and the lesser sac (dotted white arrow).

During the next 2 weeks, a gradual improvement of the patient’s clinical and laboratory findings was noted and barium upper GI series with a small-bowel follow-through study were performed. The small bowel series showed a colo-duodenal fistula between the ascending colon and the second portion of the duodenum and multiple fistulas between the jejunum and the lesser sac (Figure 5), whereas the course of the gastric fistulous tract was not identified.

**Figure 6.**
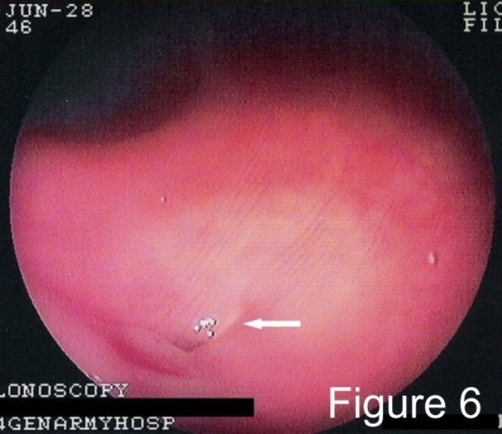
Colonoscopy. Endoscopic appearance of a fistulous tract opening in the proximal transverse colon near the hepatic flexure (continuous white arrow).

One month down the line, although the patient’s condition remained stable with partial clinical improvement under intense medical treatment, the clinical and laboratory signs of a quiescent ‘brewing’ inflammation were evident (ESR of 60 mm/1^st^ hour, CRP 60 mg/dl, low grade temperature, clammy skin, heart rate of 90 beats per minute), despite adequate control of the thyroid function (TSH 1.55 μlU/ml, FT3 1.1 pg/ml, FT4 0.84 ng/ml) and a CDAI score of 172. A second colonoscopy was performed and revealed a stenotic area in the transverse and ascending colon with erythematous, edematous, and friable mucosa with ulcers and a fistulous tract opening at the proximal transverse colon near to the hepatic flexure (Figure 6).

In the light of these findings and because of the suboptimal clinical course of the disease, despite the intense medical treatment, surgical intervention was decided to be performed. Right hemi-colectomy with ileo-transverse anastomosis, drainage of the abdominal abscess and primary closure of the upper GI tract openings (gastric, duodenal and jejunal) were performed at one stage operation. The postoperative medical management included oral steroids with gradual tapering, azathioprine, mesalamine, and antibiotics with gradual introduction of oral feeding. Still though, 3 weeks postoperatively, the markers of inflammation were high (ESR 76 mm/1^st^ hr, CRP 42 mg/l) and the patient was clammy and tachycardiac with a heart rate of 90 beats per minute despite good control of his thyroid function and a CDAI score of 105. Computed tomography (CT) scan of the abdomen and pelvis was unremarkable for intraperitoneal abscess or mass. At that point we decided to start anti-TNFa therapy with infliximab and taper-off corticosteroids, since the latter were proved insufficient in controling the disease. Infliximab infusions (5 mg/kg) were well tolerated and induced a sustained to date (36 months postoperatively) endoscopic, clinical and biochemical response. Azathioprine and mesalamine are also continued as maintenance treatment. The thyroid function maintains normal without any treatment.

## Discussion

The involvement of the upper GI tract in fistulizing Crohn’s disease with gastrocolic and duodenocolic fistulas is very rare (<1%). In most cases the fistulas arise from the diseased small or large bowel. Pain located in the epigastrium in association with nausea and vomiting are the most common symptoms, while fecal vomiting is rare [3,6,7]. Contrast imaging studies (barium enema or meal and small bowel series) and endoscopy are the most useful procedures for the diagnosis and evaluation of the fistulas [7].

In our case the predominant symptoms (fever, diarrheas, abdominal pain), were suggestive for colonic involvement, whereas the presence of nausea and non-fecaloid vomiting was not specific. Endoscopy of the upper and the lower GI identified the fistulus openings in the stomach and the duodenum, and the disease in the proximal colon, respectively. Furthermore, histologic examination of the biopsies confirmed the disease in the large bowel, while the upper GI was uninvolved except for the fistulus openings. On the other hand, the barium upper GI series with small-bowel follow-through study identified the course of the fistulus tracts.

The internal fistulas are thought to be indicative of a more aggressive subtype of Crohn’s disease and often require urgent and repeated surgical intervention. Although new immunosuppressive agents (azathioprine, 6-mercaptopurine, and infliximab) have been used for the treatment of fistulas, the majority of data refer to perianal fistulas, while controlled data for the efficacy of medical treatment in healing of internal fistulas are lacking [8-10].

The presence of abscess formation is an important issue and must be aggressively sought in patients with fistulizing Crohn’s disease. While a small abscess may be successfully treated with antibiotics, larger abscesses require intervention with percutaneous or surgical drainage [11,12]. The control of the intestinal inflammation in the area of the origin of an internal fistula and the control of the overall Crohn’s disease activity is another important issue in fistulizing Crohn’s disease. Corticosteroids, aminosalicylates, and immunosuppressive agents (azathioprine, 6-mercaptopurine, and infliximab) are usually used to treat luminal inflammatory disease. It is not uncommon, however, for these modalities to fail in controlling the inflammation and healing the concomitant abscesses or internal fistulas [8].

Our patient presented intra-abdominal fistulas involving the upper GI tract, accompanied by an abdominal abscess formation in the lesser sac. His nutritional status and his general clinical condition were quite poor on admission. We followed an aggressive medical management with hyper-alimentation (TPN), antibiotics, aminosalicylates and immunosuppressive agents (corticosteroids and azathioprine). Infliximab was not considered as the best approach both because it could complicate the intra-abdominal abscess with sepsis and because of the reported low efficacy in healing internal fistulas [13]. We could not perform percutaneous CT-guided drainage of the abscess because it was not well defined (phlegmon) and located in an area relatively difficult to access (lesser sac). The non-invasive approach was finally unsuccessful, although the patient’s general condition had improved.

In general, gastrocolic and duodenocolic fistulas represent indications for surgery. Resection of the diseased segment of the bowel containing the fistula (primary site of the disease), resection of the fistula and primary repair of the affected organ, has been proven so far to be the most effective treatment method of intra-abdominal fistulas and numerous operative approaches have been suggested [3,6,10,14]. In our patient right hemi-colectomy with ileo-transverse anastomosis, drainage of the abdominal abscess and primary closure of the upper GI tract openings (gastric, duodenal and jejunal) were performed at one stage operation, without postoperative complications.

While the surgical approach in our patient definitively cured the perforating complications of the disease (fistulas and abscess), the luminal disease in the colon remnant was still active. The on-going inflammation post-operatively, that was eventually steroid-refractory, necessitated the use of anti-TNFa, that was no longer contraindicated after the draining of the abscess, simultaneously with continuous use of azathioprine and mesalazine [15].

Thyroid involvement in Crohn’s disease has also been described in a few cases [4,5]. These reports show that thyroid disease may occur before, concurrently or after the onset of IBD [4,5]. Although there is still no clear explanation for the coexistence of thyroid disease and IBD, genetic or environmental factors may be implicated in common pathogenetic mechanisms. Moreover, the increased incidence of autoimmune diseases, including autoimmune thyroid disease, in IBD may be immune-mediated extraintestinal manifestations of IBD due to up-regulated immune responses [4,5]. Our patient presented with simultaneous occurrence of hyperthyroidism and severe fistulizing Crohn’s disease. We believe that the effective treatment of hyperthyroidism had a favorable impact on the patient’s general health condition. This also helped us to correctly associate the existence of clinical and laboratory signs of the quiescent ‘brewing’ inflammation with incomplete medical treatment of Crohn’s disease and not with thyroid dysfunction.

## Conclusion

The management of intra-abdominal fistulas in Crohn’s disease is a complex issue, which requires a multi-disciplinary approach and ‘tailoring’ of the treatment to the individual patient’s needs. Probably a sensible approach involves early surgical intervention with prior optimization of the patient’s general condition when feasible. Continuous immunosuppressive and anti-inflammatory treatment post-operatively is necessary to maintain the disease in remission and to prevent relapses. Thyroid involvement in Crohn’s disease is also possible and thyroid gland status should not be overlooked when assessing an IBD patient with active inflammation.

## Abbreviations

anti-TG: anti-thyroglobulin antibody
anti-TPO: anti-thyroperoxidase antibody
ASA: aminosalicylate acid
CD: Crohn’s disease
CDAI: Crohn’s disease activity index
CRP: C-reactive protein
CT: computed tomography
ESR: erythrocyte sedimentation rate
GI: gastrointestinal
IV: intravenous
MCV: mean corpuscle volume
PLT: platelets
TNF: tumor necrosis factor
TPN: total parenteral nutrition
TSH: thyroid stimulating hormone
WBC: white blood cell count
